# A Comparison of Measures of Boldness and Their Relationships to Survival in Young Fish

**DOI:** 10.1371/journal.pone.0068900

**Published:** 2013-07-16

**Authors:** James R. White, Mark G. Meekan, Mark I. McCormick, Maud C. O. Ferrari

**Affiliations:** 1 School of Marine and Tropical Biology, James Cook University, Townsville, Queensland, Australia; 2 Australian Institute of Marine Science, Botany Building, The University of Western Australia, Crawley, Western Australia, Australia; 3 Department of Biomedical Sciences, Western College of Veterinary Medicine, University of Saskatchewan, Saskatoon, Canada; Institute of Marine Research, Norway

## Abstract

Boldness is the propensity of an animal to engage in risky behavior. Many variations of novel-object or novel-environment tests have been used to quantify the boldness of animals, although the relationship between test outcomes has rarely been investigated. Furthermore, the relationship of outcomes to any ecological aspect of fitness is generally assumed, rather than measured directly. Our study is the first to compare how the outcomes of the same test of boldness differ among observers and how different tests of boldness relate to the survival of individuals in the field. Newly-metamorphosed lemon damselfish, *Pomacentrus moluccensis*, were placed onto replicate patches of natural habitat. Individual behavior was quantified using four tests (composed of a total of 12 different measures of behavior): latency to enter a novel environment, activity in a novel environment, and reactions to threatening and benign novel objects. After behavior was quantified, survival was monitored for two days during which time fish were exposed to natural predators. Variation among observers was low for most of the 12 measures, except distance moved and the threat test (reaction to probe thrust), which displayed unacceptable amounts of inter-observer variation. Overall, the results of the behavioral tests suggested that novel environment and novel object tests quantified similar behaviors, yet these behavioral measures were not interchangeable. Multiple measures of behavior within the context of novel environment or object tests were the most robust way to assess boldness and these measures have a complex relationship with survivorship of young fish in the field. Body size and distance ventured from shelter were the only variables that had a direct and positive relationship with survival.

## Introduction

The propensity of an animal to take a risk is often described along an axis of boldness and shyness, where high likelihood of risk-taking is defined as boldness and low likelihood is defined as shyness. This behavior is important on both ecological and evolutionary time scales. Individuals can display various levels of boldness or shyness that can influence the outcome of everyday ecological challenges, such as competition for females [1] or food [2], foraging under predation pressure [3]–[5] and habitat selection [6], [7]. Consequently, boldness and shyness can influence reproduction, survival and thus ultimately affect fitness. Boldness may have underlying physiological components and may be heritable [8]–[10], so can be subject to evolution following natural selection in subsequent generations [11].

Measurements of some aspect of behavior on the boldness-shyness axis dominate research on animal personality, termed ‘behavioral syndromes’ [12], [13]. These syndromes refer to behavioral differences among individuals or species that are consistent over time or across situations [14], [15]. Unfortunately, attempts to generalize the results of this work are hampered by a lack of common language and methodology [16], [17]. For instance, some studies have defined boldness as the tendency of an individual to move through or explore an unfamiliar space (i.e. a novel environment) [18], [19], [7], while others consider it the propensity to forage under predation risk [20] or alternatively, reaction to a novel object [21]. Additionally, researchers have used a variety of behavioral attributes to measure boldness, such as latency to emerge into a novel environment, frequency of predator inspection [3], [22], propensity to enter traps [19], or flight response to a novel object [19], [23]. These measures may have some relation to one another (i.e. correlated behavioral measures within or across certain contexts), but do not necessarily quantify the same behavioral trait [24]. Recent attempts have been made to address this issue with proposed standardized terminology [25], [26], however this has yet to be adopted universally.

The techniques used to measure boldness are almost as numerous as the studies that have assessed this trait in different taxa. Some researchers have argued that boldness should be tested in familiar, rather than novel environments [26] and to date, only a few studies have attempted to quantify behavior using multiple tests of boldness among individuals. For example, Wilson and Goden (2009) assessed individual differences in exploratory behavior, activity, and anti-predator behavior of juvenile sunfish using novel object and environment tests in the laboratory [27], while an earlier aquaria study by Brown et al. (2007) found a strong correlation between two independent assays of boldness (time to emerge into a novel environment and propensity to inspect a novel object) in a peociliid fish [28].

Due to the great variety of techniques used to quantify boldness, it remains unclear how studies compare in terms of the trait that they actually measure. Additionally, given that few assessments of behavioral syndromes have been conducted within an organism’s natural environment, it is also difficult to determine how the results of these tests predict the likelihood of real ecological consequences for the subject animals.

Clearly, there is a need to clarify the relationships among the various measures of and tests for behavior on the boldness-shyness axis on subject animals in the field. Here, we focus on this task using a tropical reef fish model. Young reef fish can be collected at the end of their larval phase immediately prior to settlement on the reef, when they are naïve to reef-based predators and behaviors learned after settlement [29]. Also, by collecting fish from a single recruitment pulse, we control for gross variations in size and age [30]. In this phase of their life cycle, reef fishes typically experience high mortality [31], with rates within the first 48 hours of benthic life averaging 57% [32], [31] but sometimes >90% [33]. The distributions that are established through differential mortality often set the pattern for abundances of juveniles and later life stages. Because experience can influence behavioral phenotypes [7], [34], [35], the use of naïve study organisms allows us to control for variation and consistency in behavior and to examine ecologically important behavioral traits at a critical ontogenetic boundary [36]. Here, we use short-term (48 hours) survival as a measure of the ecological consequences of differences in boldness, assayed using a variety of techniques. For juvenile coral reef fish, short-term survival immediately following settlement is a critical selective bottleneck for populations and is relatively straightforward to measure, making it ideal for use in our study. While our survival estimate is just one of a number of possible estimates of fitness that are ecologically relevant, because of the magnitude of mortality at this stage, the trait of survivorship is likely to be very important. For these young reef fish, we aimed to determine: 1) if different types of boldness measurements quantified a similar behavioral trait, 2) which of the commonly-used methods of assessing boldness (variants of novel object and novel environment tests) was the most closely correlated with an ecological outcome (survival), and 3) which behavioral measures were easiest to conduct *in situ* with low variability among multiple observers. Based on our previous experience with this system and study species, we predicted that novel object and environment tests would not covary in how they quantified boldness, with novel environment activity measures more likely to predict survivorship. We expected that correlations among behaviors would show that bold fish tended to be larger overall, spend more time actively foraging in ways that left them more exposed to predators, while being less reactive to any sort of novel object test than shy fish.

## Methods

### Ethics Statement

This study was carried out in strict accordance with the recommendations under James Cook University (JCU) ethics protocols and approved by the JCU Animal Ethics Committee (Permit Number: A1067). All efforts were made to minimize animal handling and stress.

### Study Site and Species

This study was conducted on the shallow reef (2–4 m depth) offshore from the Lizard Island Research Station (14°40′S, 145°28′E) on the northern Great Barrier Reef, Australia. Our study species, the lemon damsel, *P. moluccensis*, is common on Indo-Pacific coral reefs [37]. Juveniles settle from the plankton at night [38], between October and January around the time of the new moon [39], preferentially settling on live coral [40]. Larvae recruit onto the reef after approximately 20 days in the plankton, at about 11 mm standard length [41]. *P. moluccensis* has a relatively small home range [42], moving only small distances (<1 m) during the first few months after settlement [40]. Due to its high abundance, small size, rapid development, and sedentary nature, *P. moluccensis* is an ideal model organism for field and laboratory based behavioral studies [43].

### Experimental Design

#### Collection

We collected newly-metamorphosed juveniles of *P. moluccensis* using moored light traps (see small light trap of Figure 1 in Meekan et al. 2001 for design, [44]) during November 2010. Traps were anchored approximately 100 m from the nearest reef in ∼10 m of water at dusk and left overnight. Catches were emptied from the traps the next morning between 05∶30–07∶00 h. Fish collected from the traps were transported to the laboratory where *P. moluccensis* was separated from all other species and maintained in a 25 L aquarium of aerated seawater for at least 24 h to acclimatize to local conditions and reduce handling stress before experiments began. Fish were fed *Artemia* nauplii twice daily while in captivity. After acclimation, each *P. moluccensis* was placed into a clip-seal polyethylene bag containing aerated seawater and were measured for total length (to the nearest mm) with calipers, photographed, and then transported to the field in individually-labeled plastic bags. After final observations, study organisms were released unharmed on nearby natural habitat. Fish collection locations/activities and handling protocols were approved by the Great Barrier Reef Marine Park Authority (Permit Number: G10/33784.1) and JCU Animal Ethics Committee (Permit Number: A1067).

**Figure 1:**
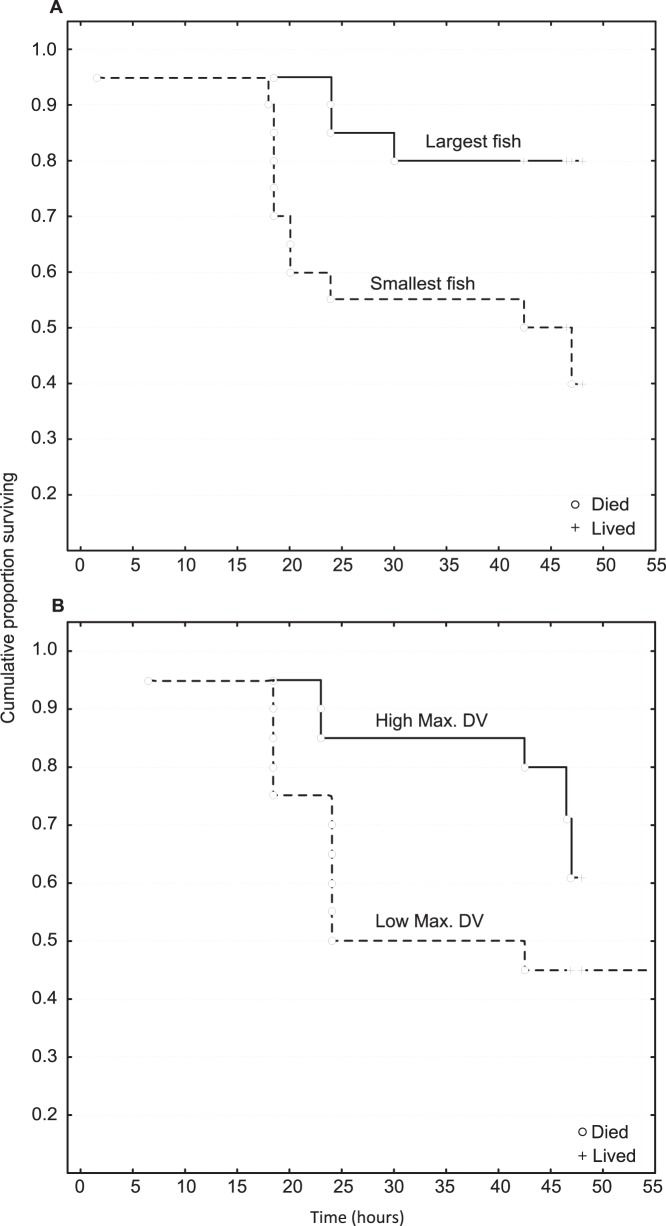
Survival over two nights in the field. Kaplan-Meier survival analysis with respect to: a) maximum distance moved and b) size (TL) of juvenile *Pomacentrus moluccensis* on patch reefs in the field. Fish were sequentially ranked for their scores on each trait and two groups (high and low ranked) of twenty fish (21.7% of total) were compared. Solid lines and dashed lines represent the two groups of highest and lowest ranked fish, respectively. Symbols represent presence or absence of individual fish during subsequent mortality surveys.

#### Observational protocol

All behavioral observations were made on individual fish in the field. Divers released a single fish onto a small patch reef (30×30×30 cm) haphazardly chosen from 35 that were constructed from live and dead pieces of the bushy hard coral *Pocillopora damicornis* on the shallow (3–4 m water depth) sand flat. *P. moluccensis* recruits occur naturally in this habitat. Reefs were deployed in rows, 5 m apart and approximately 10 m from the nearest area of natural reef. Means and ranges of temperatures did not vary among reefs (M. McCormick unpubl. data) and care was taken in reef construction to ensure that patch reefs had only very minor differences in habitat structure. Previous studies have shown that such minor variation in topographic complexity of patch reefs has no effect on behavior of young fish [36], [43]. Before introduction of the study fish, patch reefs were cleared of any resident fishes using hand nets. These were released on nearby natural reef far enough away to prevent their return (approx. 10 m). Individual study fish were then released onto their respective patch reefs and the first behavioral variable (latency to enter a novel environment; see description below) was recorded. Immediately afterwards, small wire cages (about 40×40×40 cm, 12 mm mesh size) were placed over the patch to allow the fish to acclimate to the new surroundings while being protected from predation. Cages were left a minimum of 20 min and carefully removed immediately before observations. Following established protocols, divers conducted observations from at least 1.5 m away (with the aid of a 2×magnifying glass) to avoid any effects that may have been caused by the proximity of the observer to the target fish [36], [43]. A pilot study where estimates of distance were checked against a ruler found these estimates to be within 10% of the true value.

Behavioral traits were measured for a total of 92 fish during eight periods of observation spread over 5 days. The first six of these periods (n = 59 fish) were conducted by three experienced observers, each assessing the same fish simultaneously to quantify variance in measures among observers. All subsequent observations were conducted by JRW and MGM. Data from all observation periods were used for comparisons of behavioral traits among fish and data collected by three observers was used for a comparison of variability in estimates of behavior among observers. Each behavioral test was only trialed once with individual fish because *P. moluccensis* has been shown to recognize threats after a single exposure [45], which could have altered the outcomes of some boldness measures. In general, the behavioral responses of individuals have been shown to be very stable (e.g. a coefficient of variation for 3 consecutive observations ranging from 0–0.15; repeated measures ANOVA over 15 observations across 5 days, mean intraclass correlation coefficient = 0.69) at least over the time of our relatively short experiments (Mero 2009 unpublished thesis, [36]). In both a pilot study and this experiment, we found no relationship between observed behaviors of individual fish and specific patch reefs or time of day. This suggests differences in local environmental conditions such as minor variations in habitat, light conditions and food abundance across patch reefs did not noticeably influence behaviors.

The behavior of each fish was assessed using variations of two novel-object and two novel-environment tests that were composed of 12 behavioral measures:

##### 1) Novel environment: release

After resident fish were cleared from the patch reefs, each damselfish was carefully released from the plastic bag onto the sand 10 cm from the patch reef. The amount of time it took for the fish to move onto refuge of the patch reef was termed ‘latency at release’. This was timed from the moment the fish exited the bag, to the instant it reached the edge of the reef shelter. If the individual took more than 60 seconds to move to the reef, observations were discontinued and individuals were assigned a top value (∼10% of fish).

##### 2) Novel environment: overall activity

Six behavioral measures were recorded simultaneously over a 3 min observation interval for each fish: bite rate (number of feeding strikes towards objects floating in the water column); distance moved (total distance covered (cm) during 3 min); distance ventured (the maximum distance (cm) fish moved away from their patch reef; the distance ventured from the patch (categorized as % of time spent within 0, 2, 5, or 10 cm away from the patch); and position on the reef (categorized as a cumulative proportion of the time spent at varying heights over the 3 min observation period, with the top of the patch taken as height of 1, middle of the patch a height of 0.5, and bottom a height of 0). Mean distance ventured was calculated from the sum of the proportions of time spent in each of the distance categories multiplied by the distance that each category represented. Relative height on the patch was summarized as a cumulative proportion of the time spent at varying heights over the 3 min observation period, calculated from the sum of the proportions multiplied by the height categories (0, 0.5, or 1). Estimated distances were verified with a ruler after the 3 min observation period was completed.

##### 3) Novel object: benign

Each fish was presented with a novel object (2.4×2.1×1.6 cm consistent assortment of blue and yellow Lego™ blocks, with the same blocks used for each fish) that was gently placed 10 cm away from its location. Fish were not obviously disturbed by this action. Over a 60 s observational period, minimum approach distance (cm) and a visual estimate of mean approach distance (cm) were recorded.

##### 4) Novel object: threat

The reaction of each damselfish to the thrust (∼120 cm/s over 20 cm) of an observer’s probe (pencil 13 cm long) towards them was recorded as the minimum distance from the tip of the probe (cm) before fleeing, the maximum distance traveled (cm) by the fish after the presentation of the threat, and the latency (seconds) of the fish to leave shelter of a particular part of the coral patch and return to its original location. Latency was limited to a 60 s observation time. A reaction score was quantified as a continuous variable on a 0–3 scale with 0.1 unit increments, where: **0**- hiding in refuge before or immediately after thrust and seldom emerging afterwards; **1**- retreating to refuge when scared and taking more than 5 s to re-emerge, then tentatively striking at food; **2**- retreating to refuge when scared but emerging quickly and striking at food; **3**- not hiding but continuing to explore or strike at food aggressively. The reaction score summarized the combination of overall individual behavior during the 3 min observation and reaction to the probe thrust.

### Survival

The presence of fish on reefs was monitored twice daily (between 10∶00–11∶00 and 15∶00–16∶00 h) over two days (mean 44.9 h). Previous studies have shown that any migration of newly-settled fish from patch reefs in this location is negligible (<0.007% of 300 tagged fish in 3 days) so that the absence of fish from a reefs can most likely be attributed to predation [46].

### Data Analysis

The overall variability of each behavioral measure was quantified using a coefficient of variation. The coefficient of variation and comparison of behavioral traits with survival were calculated using one score (from the most experienced observer, MGM) per fish. Behavioral responses were z-transformed to standardize differences in mean and variance while maintaining patterns of covariance.

In order to compare observers, the range of values (maximum-minimum scores) for each trait recorded by the three observers was compared across six observation periods (n = 59). Because the range values did not meet assumptions of normality, a Friedman test was used as a nonparametric alternative to one-way repeated measures ANOVA.

The influence of a single behavioral trait on survival was determined with Kaplan-Meier survival analysis and its significance with Cox’s F-Test using multiple single-predictor models. In order to highlight the influence of behaviors at either high or low extremes, the twenty highest and twenty lowest scoring fish of each trait were compared. Traits identified as significant by the Kaplan-Meier test were further compared using phenotypic selection gradient analysis [47] as a more explicit test of the relationships between single and combinations of traits on fitness. This test was used to identify behavioral traits that best predicted survivorship, while accounting for direct and indirect selection. First, behavioral variables were z-transformed (standardized). Then, logistic regression was used to regress the standardized values, their squared terms, and the cross-products of the pairwise combinations on relative fitness (whether an individual lived or died, divided by average fitness of the population) to estimate directional, stabilizing, and correlation selection gradients, respectively [47], [34].

Relationships between behavioral traits were analyzed using Pearson’s product moment correlation. The statistical effect value (r) associated with these correlations are simply used as potential indicators of the strength of relationships rather than indicators of biological significance. However, sequential Bonferroni adjustments are included to account for multiple testing (Type I) errors.

Confirmatory factor analysis, a form of structural equation modeling [48], was used to determine the structure of a combination of behavioral measures used to assess boldness for the population during a 48 hr post-settlement period. We followed the proposed framework established by Dingemanse et al. (2010) for using structural equation modeling (SEM) to compare hypothesized patterns of behavioral covariance. Eight alternative models formulated *a priori* (as described below) for boldness syndrome structure were separately assessed and the relative fit of each model was compared. Models were compared using Akaike’s Information Criterion (AIC), which was calculated from model discrepancies (Ĉ) estimated by maximum likelihood using Bollen-Stine bootstrapping (2000 bootstraps). AIC values compare the fit of a model to data while rewarding parsimony, with lower values indicating greater model support [49], [50]. Models were compared by AIC differences (ΔAIC) relative to the model with the lowest AIC value, with ΔAIC values greater than two suggesting less support [51]. The maximum convergence limit for data to fit to models was set at 50 iterations.

In order to increase parsimony of the structural equation models, the most similar behavior responses were combined into composite variables by extracting their factor scores using factor analysis. Distance ventured and maximum distance ventured were combined into a new variable termed ‘Exposure’. Minimum and average distances to Lego blocks were combined to form the new variable ‘Benign response’, while minimum and maximum distances to the threatening object (probe) formed the new variable ‘Flight response’. Rather than the traditional method of using factors with eigenvalues greater than one, parallel analysis was used to determine the number of factors to be extracted (using permutations of 1000 parallel generated datasets) as outlined in Budaev (2010). With the correct number of factors determined by the parallel analysis, factor scores were calculated using principle axis factoring with Varimax rotation and the regression method [52].

Prior to SEM analysis, Bartlett’s test of sphericity and the Kaiser-Meyer-Olkin (KMO) index were calculated for the dataset. The sphericity test determined if the behavioral variance-covariance matrix differed from random [50], while the KMO index compared observed correlations and partial correlations among original variables [52]. In our data, the matrices differed from random (χ^2^_28_ = 118.40, P<0.001). The KMO values were above the 0.5 acceptable threshold [52] with KMO = 0.52. However, the efficacy of the KMO test for a confirmatory factor analysis with a single latent factor (as used in this study) is unknown [50] and both tests are unlikely to be necessary for simple models with few observed variables [53].

**Figure 2:**
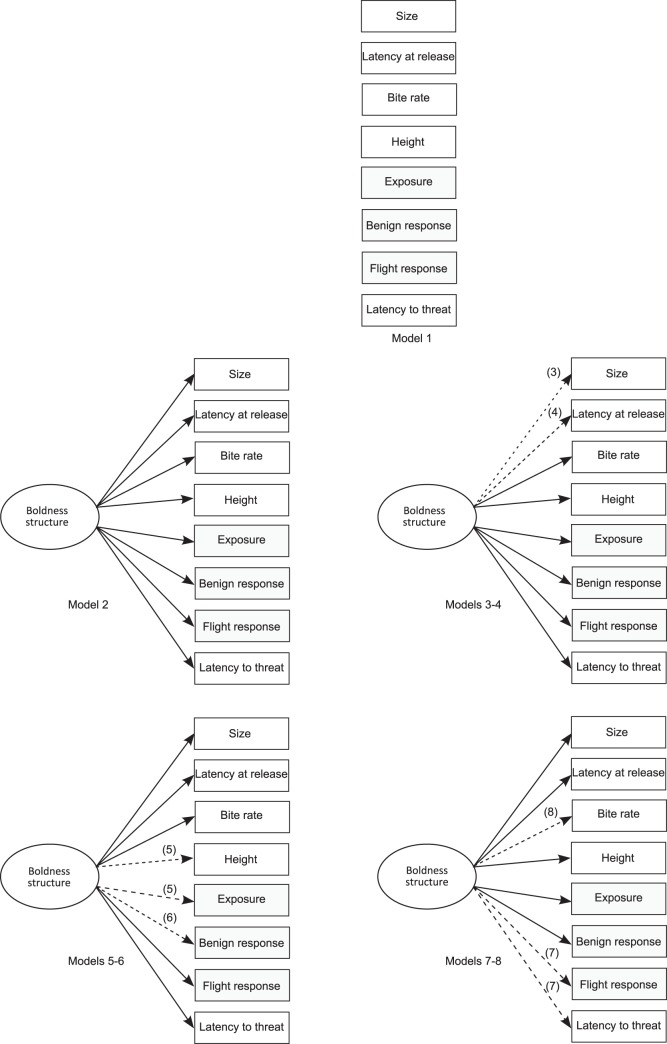
Eight models of boldness syndrome structure developed based on *a priori* hypotheses of boldness structure. Model 1 represents behavioral independence. Model 2 represents a domain-general model of syndrome structure while models 3–8 are more constrained, representing different types of boldness tests. The measured behaviors are represented in rectangular boxes, with shaded boxes representing composite variables. Underlying causal connections (latent variables) resulting in boldness structure are represented in ovals [48]. In order to save space, multiple models are presented with alternative structures denoted by dashed lines labeled with model number (e.g. model 3 excluded size, as denoted with a dashed line labeled 3).

Eight *a priori* hypotheses of boldness structure were considered based on the different types of boldness tests in behavioral syndrome literature (models 1–8, Fig. 2). Model 1 was the null model, where there was an absence of covariance and behavioral responses varied independently [54]. Model 2 represented a domain-general model of boldness structure, where all types of novel environment and novel object tests were linked via an underlying factor. Models 3 and 4 represented a domain-general model where size and latency at release, respectively, were considered contextually different from the rest of the behavioral responses. Model 5 considered foraging and height contextually different. Model 6 removed the benign response from the other boldness measures. Model 7 removed the threatening novel object measures: flight response and latency to threat. Model 8 considered bite rate contextually different from other activity, novel object, and novel environment tests.

Because models were built on *a priori* hypotheses, models 2–8 were compared against the model of no boldness syndrome structure (model 1) to quantify the amount of variation explained by the different models. This was done by calculating D_x_, which represented the proportion of variation in the behavioral variance-covariance matrix explained by each model, relative to the null model [55], [50]. D_x_ was calculated as: D_x_ = 1- Ĉ_x_/Ĉ_null_ where Ĉ_null_ was the discrepancy for the null model (i.e. model 1, Fig. 2) and Ĉ_x_ was the discrepancy for other hypothesized models (i.e. models 2–8, Fig. 2). D_x_ is interpreted similarly to an R^2^ value [50].

Statistical analyses used SPSS version 20.0 (SPSS Inc., Chicago, IL, U.S.A.). Structural equation models were constructed using AMOS version 20.0 (SPSS, Inc.).

## Results

### Variability of Behaviors

Most traits showed high variability among individuals (Table 1), which allowed one or more traits to affect post-settlement mortality. Coefficients of variation ranged between 8–82% for most measures, with the exceptions of latency at release, time budget and escape latency to a probe thrust, which all had CVs over 100% of mean values. Latency at release had the highest CV (167%), but this was skewed due to a small number of fish (9 of 92 fish) that did not move to patch reefs within the 60 s observation period. The CV reduced to 102% when these slow-to-respond fish were excluded from the data set. The time budget had high CVs since few fish remained motionless or did activities other than feeding. Some fish (7 of 92) remained hidden within the refuge of the patch reefs after the probe thrust, skewing the CV for this measure.

**Table 1:** Summary statistics for various measures of novel object or novel environment tests of the Lemon damsel (*Pomacentrus moluccensis*).

Variable	N	Mean	SD	CV (%)	Mean Inter-Observer SD/Equivalent in units
*Physical character*					
Size (cm)	92	1.3	0.1	8	N/A
*Novel environment: release*					
Latency at release (s)	92	17.1	28.5	167	1.0/2
*Novel environment: activity*					
Bite rate	92	26.7	15.1	56	8.1/16
Distance moved (cm)	92	17.5	14.4	82	8.4/15
Distance ventured (% time index)	92	1.8	0.9	52	0.6/1
Max. distance ventured (cm)	92	3.3	1.8	55	1.8/3
Position on reef (height index)	92	2.5	0.7	26	0.1/0.2
*Novel object: benign*					
Minimum distance to Legos (cm)	92	4.2	1.8	44	1.0/2
Mean distance to Legos (cm)	92	7.2	2.7	37	1.2/2
*Novel object: threat*					
Minimum distance to threat (cm)	92	3.2	2.2	69	0.7/2
Max. distance travelled from threat (cm)	92	5.3	1.8	34	1.6/3
Latency to threat (s)	92	14.2	18.3	129	2.4/4
Threat test (0–3 score)	92	1.7	0.6	37	0.4/0.8

Mortality was monitored for at least two nights in the field (mean 44.9 h). A total of 41.8% of all fish disappeared from reefs and were assumed to have died (Table 2). Of these, 84% died within the first 24 h, typically at sometime between the last observation in the afternoon and the next observation the following morning.

**Table 2:** Survival (%) of newly settled Lemon damsel (*Pomacentrus moluccensis*) on patch reefs.

Field trial	Trial duration (h)	N	Survival (%)
1	47	9	60
2	47	7	57
3	42.5	9	67
4	42.5	12	67
5	42.5	7	43
6	42.5	14	43
7	48	14	64
8	47	20	65
Mean or total	44.9	92	58

### Variability among Observers

The threat test was the only measure that showed significant variability among observers across trials (χ^2^(5) = 12.72, p = 0.026). There was no pattern of improvement in observer consistency over time, with the variability in trials 1, 4, and 6 lower on average than trials 2, 3, and 5. The variability in threat test scores among observers ranged from a 0.5 to 1.0 difference (on 0–3 scale).

Other behavioral measures did not differ significantly among trials and observers, suggesting no major improvement or decline in observer consistency. Most variables had a low level of observer variance (Table 1), with a difference of only 1–3 cm or 1–3 seconds (∼3% of maximum observation time). However, distance moved had a relatively large variance, with observers disagreeing by an average of 12 cm in most trials, although this improved to 5 cm by the end of the study. Estimates of bite rate were moderately variable but improved with time, with the average difference ranging between 6–22 strikes. Because of the high and inconsistent inter-observer variability in measures of the threat test and distance moved, these measures were omitted from subsequent analysis.

### Individual Behavioral Traits and Survival

The ability to discriminate survivors from non-survivors on the basis of a single behavioral trait was poor. However, Kaplan-Meier survival analysis showed maximum distance ventured (F_12,22_ = 2.42, p = 0.035) and initial size (F_8,22_ = 3.72, p = 0.007) were good predictors of survival (Figures 1 a & b). Larger fish and those willing to venture further from the reef had better survival rates. Bag latency at release was suggestive of a trend (F_16,28_ = 1.97, p = 0.056), with fish that quickly moved to the patch reef having lower average mortality.

The phenotypic selection analysis showed a significant relationship between the behavioral traits and relative fitness (), however this model accounted for a relatively low amount of the variation with Cox & Snell R^2^ = 0.096 (Table 3). Overall, larger fish survived better, with size as the only variable identified as significant directional (β = 0.469, p<0.05) and stabilizing (β = 0.234, p<0.05) selection gradients, even though the size range was only 1.1–1.6 mm total length. No other directional, stabilizing, or correlational selection gradients were found to be significant. The model was adequate and predicted 63% of the responses correctly.

**Table 3:** Directional, stabilizing and correlational standardized selection gradients (β) from logistic regression.

	β	SE	*P*- value	β *avggrad*
Size	0.469	0.240	0.050	0.170
Latency at release	−0.418	0.234	0.074	−0.152
Max. DV	0.072	0.229	0.754	0.026
Size^2^	0.234	0.120	0.050	0.085
Latency at release^2^	−0.209	0.117	0.074	−0.076
Max. DV^2^	0.036	0.115	0.754	0.013
Size * Latency at release	0.256	0.280	0.360	0.093
Size * Max. DV	0.100	0.230	0.665	0.036
Latency at release * Max. DV	0.232	0.206	0.261	0.084

Model , *P* =  0.026, Cox & Snell R^2^ = 0.096.

### Correlations among Behavioral Traits

There were two significantly correlated relationships between behavioral traits (Table 4). Bite rate had a high positive correlation with exposure. Bite rate was also moderately negatively correlated with latency to a threat. This general lack of correlation suggests that each variable is quantifying a different aspect of behavior or space use.

**Table 4:** Phenotypic correlations between seven behavioral traits for Lemon damselfish.

†Behavior	Relative fitness	Size	Latency at release	Bite rate	Height	Exposure	Benign response	Flight response	Latency to threat
Relative fitness	–	0.25*	−0.24*	0.00	0.04	0.18	0.09	−0.05	−0.12
Size		–	−0.22*	0.11	0.21*	−0.26	−0.05	0.10	0.08
Latency at release			–	−0.13	−0.15	0.01	0.14	−0.09	0.20
Bite rate				–	0.16	0.61***	−0.12	−0.01	−0.35***
Height					–	−0.05	−0.31**	0.19	−0.16
Exposure						–	0.11	0.13	−0.24*
Benign response							–	0.25	0.30**
Flight response								–	0.15
Latency to threat									–

### Structure of Multiple Behavioral Traits

There was equal support for models in which response to the benign novel object (model 6, ΔAIC = 0; Table 5), size (model 3, ΔAIC = 0.10; Table 5), latency at release (model 4, ΔAIC = 0.29; Table 5) varied independently of other behavioral measures and also for the model in which all measures were included (model 2, ΔAIC = 0.85; Table 5). These models explained approximately 51% of the variance-covariance matrix variation in behavior (Table 5). In summary, four models fit the data equally well and accounted for about half the total variation.

**Table 5:** Model comparison results for confirmatory factor analysis.

Model (x)	Ĉ (discrepancy)	k	AIC	ΔAIC	D_x_
6	58.76	15	88.76	0	0.51
3	58.87	15	88.87	0.10	0.50
4	59.05	15	89.05	0.29	0.50
2	57.61	16	89.61	0.85	0.52
5	92.00	14	119.99	31.23	0.23
8	91.66	15	121.66	32.89	0.23
1	118.8	8	134.8	46.04	0

Structural equation models (SEMs) were evaluated based on difference in Akaike’s information criterion (AIC) values. Small values represent an increased parsimony-informed fit to the data. AIC values were calculated based on the discrepancy between the statistical model for a hypothesis (Ĉ) and the number of parameters (k). D_x_ values represent the proportion of the variance explained by the focal model relative to null expectations of no boldness structure. D_x_ can be interpreted as analogous to R^2^. Unlisted models were those where the data did not converge within 50 iterations.

**Figure 3:**
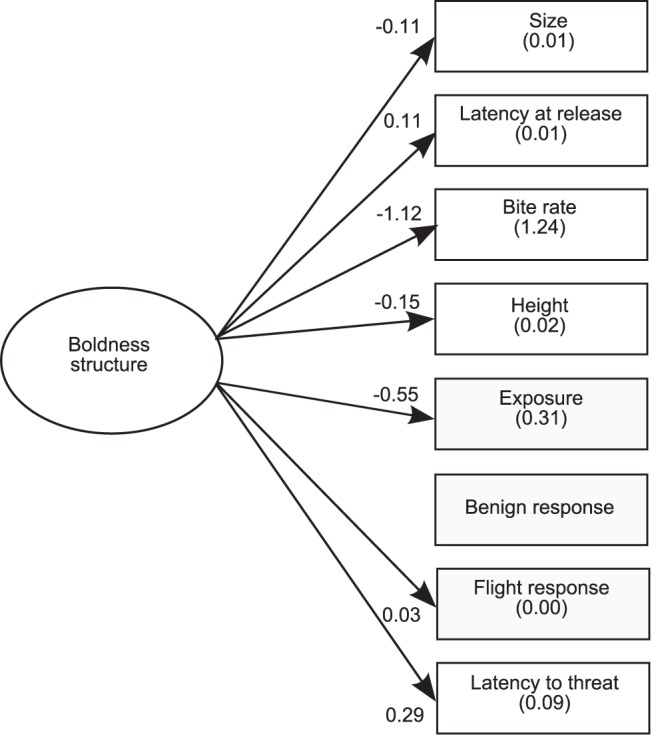
Best fitting structural equation model (SEM). This SEM shows how behaviors were related within the best fitting model for damselfish. Numbers in parentheses are variances of the different behaviors explained by the SEM structure (R^2^) for ‘model 6′ (see Fig. 2). Numbers associated with arrows are standardized factor loadings for the effects of the underlying boldness structure on a particular behavior. These represent how behavioral responses are predicted to change based on changes to the underlying boldness structure (e.g. a shift of 1 SD along the distribution of boldness structure for the population would result in a 0.15 SD decrease in height).

The behavioral patterns were best explained by models that showed a similar pattern in variable loadings. Path coefficients for the best fit models (models 2–4, 6) all had negative loadings for bite rate, exposure, size and height and positive loadings for latency at release, latency to threat, benign novel object and flight responses (Fig. 3). Loadings with the same sign imply an unknown proximate factor or factors that affect the expression of behaviors in the same manner [50]. The SEM structure explained a high amount of variance in data sets for bite rate and exposure behaviors, suggesting these measures were better suited to assess boldness of juvenile fish in the field.

## Discussion

### Individual Behavioral Traits and Survival

Single behavioral traits had limited ability to predict survivorship for our model species. Those fish that were larger or were willing to venture further from the edge of patch reefs had greater survivorship during this critical phase of the life cycle; a conclusion supported by studies of intra- and inter-specific behavioral interactions at this life stage [56], [57]. There was a strong (though non-significant at p = 0.056) trend for fish that moved quickly to patch reefs when released to survive better than those that were slow to travel to the reef. Phenotypic selection analysis suggested only size had a significant effect on survivorship and that combinations of behavioral measures did not influence survival. Size and condition at settlement has previously been shown to be important for survival [58], [59], with larger fish often having greater survivorship [60]. However, this pattern is not consistent at all times and places, with some studies showing that newly-settled individuals that were larger suffered higher mortality than smaller fish in some cases [43], [61]. Additionally, earlier work has found no links between foraging behaviors and selective mortality at settlement [43], or a positive correlation between distance ventured from reefs and mortality [36]. Such differences in outcomes of studies may simply be a reflection of the temporal or spatial variability in predator/prey abundance [62], [63] or a predator’s individual preference of prey species [64]. These complex relationships between predator/prey abundance and predator behaviors could be a major driving force in shaping individual variation in the prey’s behavior and ultimately, survival in the population. For example, Holmes & McCormick (2009) have shown that one of the major predators on newly-settled damselfish, *Pseudochromis fuscus*, which is common in shallow reefs adjacent to our patch reefs [61], [65], preferentially targets larger recruiting fishes. If *P. fuscus* was more abundant in previous years, or selectively targets certain species [66], then spatial and temporal differences in the relationship of size or behavioral traits with mortality would be expected.

We used short-term (over 2 nights) survival as an ecologically relevant measure of the consequences of behavioral decisions although other measures of fitness (e.g. long term survival, reproductive output, offspring quality, etc.) or some other aspect of an animal’s ecology could be used as an equally valid trait against which behaviors could be compared. Indeed, the different measures of boldness might vary in relevance depending on the trait against which they are measured and ontogenetic stage [2]. The high and selective mortality that normally occurs during the settlement transition for organisms with complex life cycles such as fishes makes the short term mortality measured in the present study, and the behavioral correlations explored, ecologically relevant.

### Correlations among Behavioral Traits

The limited number of correlations among behaviors found in our study suggests that the behavioral variables we assessed measured slightly different aspects of boldness and were not interchangeable. The positive relationship between the composite variable ‘Exposure’ and bite rate was expected because juvenile fish tend to actively swim and explore the vicinity of their habitat while foraging. Fish that had higher bite rates also tended to quickly resume feeding after being threatened with a probe. With size being the principal predictor of short-term survival, one viable strategy would be for these fish to prioritize behaviors that maximized growth rates. By growing quickly, juveniles would escape gape-limited predators and better compete for space and resources. In this case, it would be advantageous for juvenile pomacentrids to quickly learn to recognize and ignore false threats, a trait that is a feature of these fishes [45].

### Structure of Multiple Behavioral Traits

Multiple SEM models could be fitted to the data for juvenile lemon damselfish. This suggests that there was considerable variability in the expression of boldness among individuals at the same life stage, in this case within the first few days of settling to the coral reef environment. Having a relatively adaptable expression of boldness at this time may allow individuals to properly assess and deal with the risks associated with the large assortment of predators that preferentially target fish recruits.

The use of a wild-caught population of juvenile fish rather than laboratory-bred individuals may account for a lower value for overall model fit (D_x_ = 0.51) compared to similar studies [50]. Previous work has shown similar species of juvenile damselfish are highly flexible in their behavioral responses across different situations (White et al. in review). Relatively large individuals also had relatively high bite rates and spent more time near the top of the reef (greater height) while being relatively quick to exit the bag at release, were more exposed, and less reactive to novel objects. This was in agreement with our predictions on how boldness would be structured. However, contrary to our predictions, novel object and novel environment tests did not vary independently, with the fit of the data lending equal support to the unrestricted domain general model (model 2). All measures were considered to be behavioral responses that were contextually similar in regards to boldness structure. In other words, all measures accounted for the structure of boldness.

### Variability among Observers

Variability among observers measuring the same trait did not decline or increase over time for most behaviors, with the exception of the threat test. Variation in this measure increased during the study, probably reflecting the subjective nature of the measure, at least when multiple observers were involved in the work. Measures of bite rate, escape distance from a probe thrust and minimum distance from a probe thrust all showed some signs of reduced variation among observers over time. Observer variation in observed bite rate was initially high, but was reduced to acceptable levels after limited training. Overall, generation of consistent and accurate measures of distance moved and reaction to the threat test proved difficult when multiple observers were involved, however the recording of behavior using high resolution cameras may offer a means to further reduce this source of variation in these measurements.

### Conclusion

Although we measured 12 behavioral variables, only one (distance from shelter) predicted short-term survival. Fish size (a physical character) was the most influential in determining survival. In the past, most studies have considered boldness as a binary trait that was that could be quantified with a single variable. However, our study suggested that multiple measures of behavior and habitat use were necessary to adequately quantify boldness in our study species, because all quantified slightly different and largely uncorrelated aspects of behavior. Additionally, our multivariate analysis suggested that both novel object and environment tests were related via some underlying causal factor to boldness structure, but the lack of correlations suggested that these behavioral measures were not interchangeable. For our study animal, a tropical reef fish, we argue that most of the behavioral variables measured that required little to no interaction with the study subject gave a good overall insight into boldness structure. Boldness measures that involve interaction (e.g. presentation of novel objects), while correlated with another measure (bite rate), provided only a small amount of additional predictive value with regards to boldness structure of the fish. Also, due to the ability of *P. moluccensis* [45] and other juvenile fishes [67], [68] to learn rapidly, novel object tests may be less repeatable once fish have acclimated toward the stimuli [69]. We suggest that novel object tests may engender responses that have little relevance to the environments in which naïve young fish find themselves after settlement, so that the results may have no bearing on the likely behavior of individuals in response to natural predators, at least in the first few days after settlement. While our results show novel environment and object tests both give insight into boldness structure, the repeatability and ecological relevance should be considered when selecting the most appropriate boldness measure for a study organism.
